# Moderate Exercise Mitigates Diabetes-Induced Hippocampal Dysfunction in Aged Rats

**DOI:** 10.3390/ijms27146450

**Published:** 2026-07-20

**Authors:** Saltuk Bugra Baltaci, Gözde Acar, Elif Gulbahce-Mutlu, Ayyuce Kumas, Ayşenur Feyza Bayiroglu, Rasim Mogulkoc, Abdulkerim Kasim Baltaci

**Affiliations:** 1 Research Institute for Health Sciences and Technologies (SABITA), Department of Physiology, Medical Faculty, Istanbul Medipol University, 34810 Istanbul, Türkiye; bugrabaltaci@icloud.com; 2 Department of Physiology, Medical Faculty, Selçuk University, 42130 Konya, Türkiye; gzdcr67@gmail.com (G.A.); ayyucekumas.97@gmail.com (A.K.); mogulkoc@selcuk.edu.tr (R.M.); 3 Department of Medical Biology, Medical Faculty, Konya Chamber of Commerce (KTO), Karatay University, 42020 Konya, Türkiye; elif.mutlu@karatay.edu.tr; 4 Department of Physiology, Medical Faculty, Bandirma Onyedi Eylul University, 10200 Bandırma, Türkiye; feyza-bayiroglu65@hotmail.com

**Keywords:** diabetes, aging, hippocampus, exercise, neuroplasticity, NZF-2b

## Abstract

This study investigated the effects of moderate chronic exercise on hippocampal integrity, motor learning, and neuroplasticity markers in diabetic aged (16-month-old) female Wistar rats. Forty Wistar rats were divided into four groups: Control (*n* = 10), Exercise Control (*n* = 10), Diabetes (*n* = 10), and Diabetes + Exercise (*n* = 10). Diabetes was induced via streptozotocin (40 mg/kg, i.p.), while exercise groups performed 45 min daily treadmill sessions for four weeks. Motor coordination was assessed using the rotarod test. Hippocampal Nogo-A, KLOTHO, and NZF-2b gene expressions were analyzed by RT-PCR, and oxidative stress markers (MDA, GSH) were measured by ELISA. In the diabetes group (G3), Nogo-A and MDA levels significantly increased (*p* < 0.05), while KLOTHO, NZF-2b, and GSH levels decreased (*p* < 0.05). Chronic exercise reversed these pathological changes by decreasing Nogo-A and MDA (*p* < 0.05) and increasing KLOTHO, NZF-2b, and GSH (*p* < 0.05). Behaviorally, the diabetes group (G3) showed the lowest rotarod performance (*p* < 0.05), which significantly improved with exercise (*p* < 0.05). Findings suggest that diabetes and aging impair neuroplasticity-related gene expression and increase oxidative stress in the hippocampus. Chronic exercise (G2 and 4) exerts neuroprotective effects by mitigating these damages. Specifically, the upregulation of NZF-2b indicates that exercise may support neuroplasticity through transcriptional regulation, offering a preventive strategy against diabetic hippocampal degeneration.

## 1. Introduction

The relationship between aging, diabetes, and the hippocampus is complex and interconnected; diabetes, particularly type 2 diabetes (T2DM), accelerates brain aging and impairs hippocampal function, which is critical for cognition and memory. Indeed, this relationship between aging, diabetes, and the brain has led to the theory of advanced brain aging in diabetic patients [1,2]. Normal aging is characterized by declines in hippocampal structure and function, including decreased synaptic density, interneuron loss, and impaired synaptic plasticity. These changes are associated with cognitive decline, particularly in memory formation and recall. Aging also involves brain atrophy and decreased neurogenesis in the hippocampus, contributing to decreased cognitive performance [1,2]. Both type 1 and type 2 diabetes exacerbate many of the hippocampal changes seen in aging. Experimental models and clinical studies have shown that diabetes reduced hippocampal neurogenesis, dendritic remodeling, and synaptic loss, increased neuronal apoptosis, impaired glucose and insulin regulation in the brain—vital for hippocampal function—leading to impaired neuroplasticity affecting learning, memory, and emotional regulation [1,2,3,4]. These changes contribute to cognitive impairments and an increased risk of depression; depression itself is a risk factor for diabetes, suggesting a reciprocal relationship [4]. The overlap between diabetes-induced and age-related hippocampal changes suggests that diabetes may exacerbate cognitive decline and increase vulnerability to neurodegenerative diseases such as Alzheimer’s disease [5]. The reports presented above ultimately highlight the importance of glycemic control and potential pharmacological interventions to protect hippocampal function and thus cognitive health in diabetic and aging populations [1,2,3,4]. Consequently, the hippocampus is a critical brain region affected by both aging and diabetes, and metabolic dysfunction is closely associated with neurodegeneration and cognitive impairment [1,2,3,4,5].

Nogo-A, a high molecular weight membrane protein found abundantly in oligodendrocytes in the central nervous system, is a member of the Reticulon-4 (RTN4) protein family [6]. First identified as a myelin-bound axonal growth suppressor, Nogo-A has been shown to strongly inhibit axonal regeneration in adult mammals [6,7]. In this context, suppression or blockage of Nogo-A function supports axon elongation following nervous system injury and enhances functional recovery [6,7]. The physiological role of Nogo-A is not limited to the inhibition of axon regeneration. It also plays a role in the regulation of synaptic plasticity, neuronal junction stability, and neurotransmitter balance in both the developmental and adult central nervous systems [8]. In elderly individuals, Nogo-A is synthesized in the hippocampus by both neuronal cells and oligodendrocytes. This protein participates in myelination processes and acts as a limiting factor for axonal regrowth and neuronal plasticity [9,10]. It has been reported that Nogo-A protein levels increase in the hippocampus during the normal aging process, and this increase becomes more pronounced in Alzheimer’s disease (AD) cases. This increase has been found to be associated with beta-amyloid accumulations observed in senile plaques [9,10]. In contrast, mRNA levels of the Nogo gene are observed to selectively decrease in the hippocampus as aging progresses [9,10].

Klotho is a protective protein widely expressed in the brain, including the hippocampus, with important roles in antioxidant defense, synaptic integrity, and cognitive function [11,12]. Both animal and human studies have shown that α-Klotho expression in the hippocampus decreases significantly with aging [13]. Genetic deficiency of Klotho plays a critical role in brain aging in experimental animals, leading to early deterioration of cognitive performance, tissue damage, and hippocampal cell apoptosis [12]. Decreased Klotho levels in cerebrospinal fluid are linked to neurodegenerative processes in both aging processes and Alzheimer’s disease [13]. Conversely, overexpression of α-Klotho in hippocampal neurons of aged mice improves memory performance and synaptic transmission [13].

**Scheme 1:**
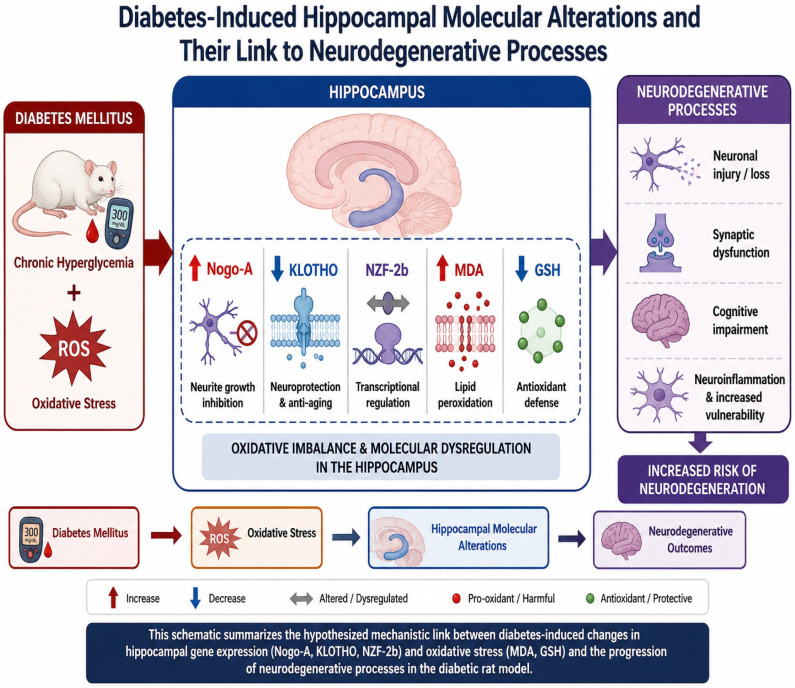
Diabetes-induced hippocampal molecular alterations and their link to neurodegenerative processes.

Brain-specific neural zinc finger transcription factor-2b (NZF-2b) is known to contribute to various functions, including neuronal differentiation, synaptic function, and survival, by regulating gene expression in the developing and adult nervous system [14,15]. NZF-2b has also been identified in mouse models and shown to influence brain behavioral responses through different neuroplasticity pathways [16]. Animal studies and human epidemiological data in type 2 diabetes models show that hyperglycemia reduces neurotrophic factors such as BDNF and impairs synaptic plasticity [17]. Chronic exercise, especially for periods of 10 weeks or more, has been reported to reduce neuroinflammation and increase neurogenesis in the hippocampus in mouse models with type 2 diabetes [18]. There are no reports investigating the relationship between chronic exercise and changes in NZF-2b expression in the aged brain under diabetic hyperglycemia. Based on the information presented above, it can be suggested that chronic exercise may potentially affect NZF-2b gene expression in the diabetic elderly brain (Scheme 1).

The Rotarod performance test is a widely used behavioral analysis method to quantitatively assess motor coordination, balance, musculoskeletal function, and motor learning capacity in experimental animals [19]. Based on measuring the time an animal remains on a rotating cylinder, this test exhibits high sensitivity in detecting the effects of neurodegenerative processes, aging, and metabolic disorders on the motor system [20]. Peripheral neuropathy, muscle atrophy, or central nervous system (CNS) modulations, particularly seen in diabetic senile female rats, are characterized by a significant decrease in Rotarod performance, which constitutes an important biomarker for disease progression or the effectiveness of therapeutic interventions. Furthermore, while learning curves obtained across successive trials shed light on motor adaptation capacity, the motor performance profile created by the Rotarod test allows for the interpretation of results in cognitive tasks independently of motor deficiencies [19,21].

The primary aim of this study was to evaluate the molecular and functional effects of aging and diabetes on the hippocampus and to investigate the potential neuroprotective effect of chronic exercise against these adverse changes. To this end, the expression levels of Nogo-A and Klotho proteins, associated with neuroplasticity and neurodegeneration, as well as NZF-2b, which plays a role in neuronal differentiation and the regulation of gene expression, were examined in the hippocampus of diabetic aged female rats. Furthermore, the histological and molecular level assessment of whether chronic exercise reduces aging and diabetes-related hippocampal tissue damage was conducted. To determine the functional counterpart of the molecular findings, motor coordination and balance performance were analyzed using the rotarod test, and the obtained behavioral outcomes were correlated with hippocampal molecular changes.

## 2. Results

**Figure 1:**
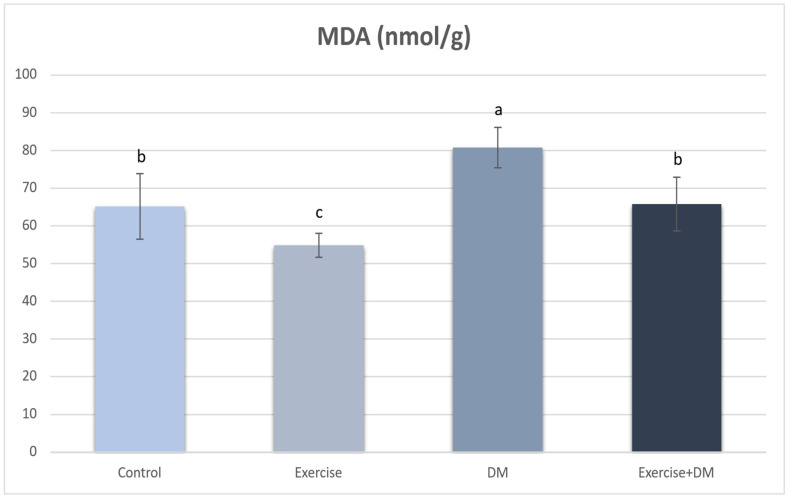
Hippocampal MDA levels. Results are presented as mean ± SEM. There are statistically significant differences between groups labeled with different letters (*p* < 0.05; a > b > c). The unit is presented as nmol/g tissue. *p*-Values MDA: Control-Exercise: 0.0434; Control-DM: 0.0049; Control-DM + Exercise: 0.9988; Exercise-DM: <0.0001; Exercise-Exercise + DM: 0.0471; DM-Exercise + DM: 0.0073. The sample size was identical across all experimental groups (*n* = 10 animals per group) for all assays.

**Figure 2:**
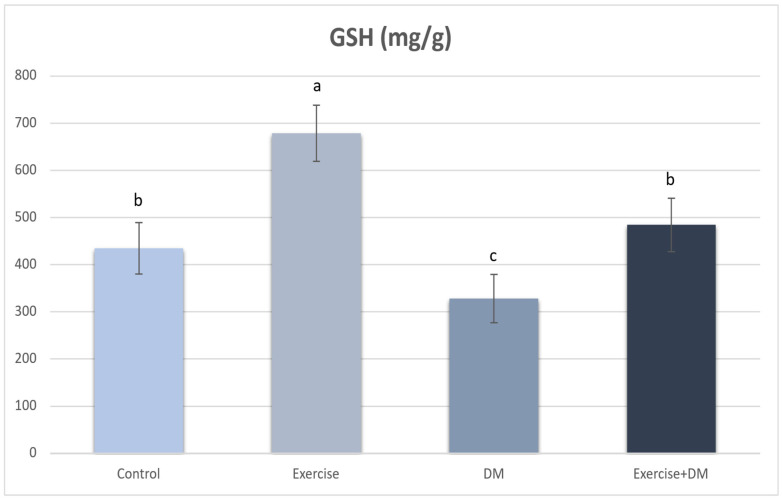
Hippocampal GSH levels. Results are presented as mean ± SEM. There are statistically significant differences between groups labeled with different letters (*p* < 0.05; a > b > c). The unit is presented as mmol/g tissue. *p*-Values GSH: Control-Exercise: 0.0001; Control-DM: 0.0438; Control-Exercise DM: 0.4743; Exercise-DM: 0.0001; Exercise-Exercise + DM: 0.0041; DM-Exercise + DM: 0.0258. The sample size was identical across all experimental groups (*n* = 10 animals per group) for all assays.

Statistical comparison of the data obtained from the experimental animals used in the study [Group 1 (G1, Control), Group 2 (G2, Exercise), Group 3 (G3, DM), Group 4 (G4, Exercise + DM)] is presented below. In the hippocampal tissue analyses, the highest malondialdehyde (MDA) levels and the lowest glutathione (GSH) levels were observed in the Diabetes group (G3) (*p* < 0.05). Chronic exercise administered to diabetic rats (G4) significantly improved oxidative stress parameters compared with the G3 group, as evidenced by decreased MDA levels and increased GSH levels (*p* < 0.05). While the improving effect in G4 reached the values of the G1 group, it did not fully reach the values of the G2 group (*p* < 0.05; Figure 1 and Figure 2).

**Figure 3:**
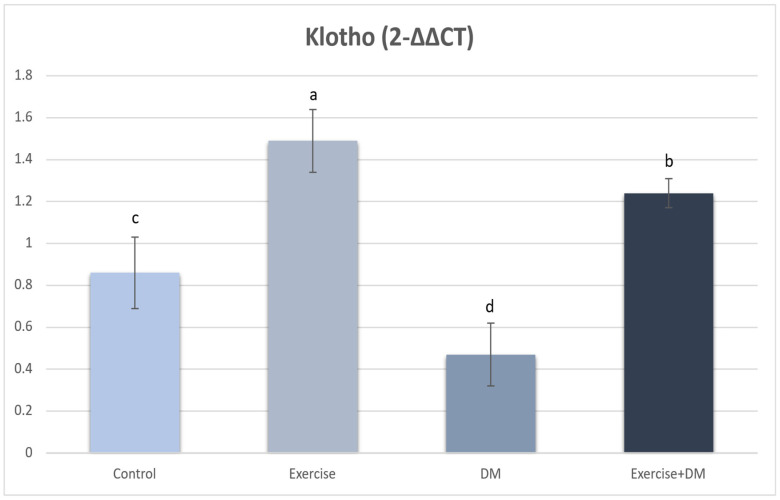
Hippocampal *Klotho* gene expressions. Results are presented as mean ± SEM. There are statistically significant differences between groups labeled with different letters (*p* < 0.05; a > b > c > d). GAPDH was used as an endogenous control. *p*-Values KLOTHO: Control-Exercise: 0.000; Control-DM: 0.000; Control-Exercise + DM: 0.000; Exercise-DM: 0.000; Exercise-Exercise + DM: 0.000; DM-Exercise + DM: 0.000. The sample size was identical across all experimental groups (*n* = 10 animals per group) for all assays.

**Figure 4:**
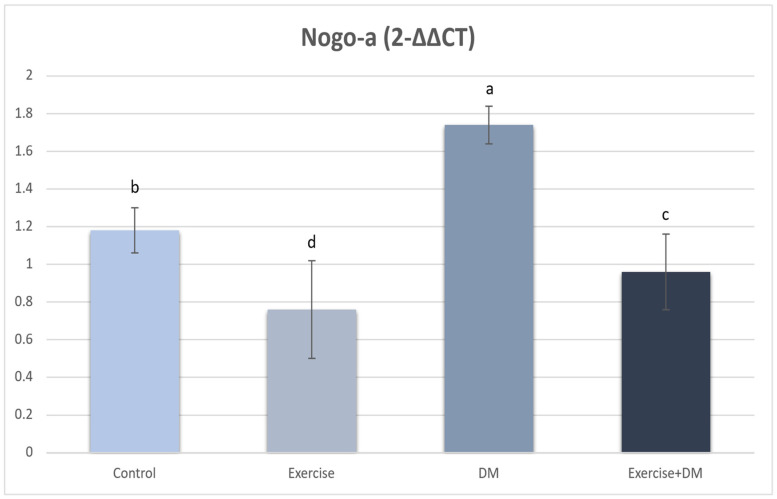
Hippocampal *Nogo-a* gene expressions. Results are presented as mean ± SEM. There are statistically significant differences between groups labeled with different letters (*p* < 0.05; a > b > c > d). GAPDH was used as an endogenous control. *p*-Values NOGOA: Control-Exercise: 0.036; Control-DM: 0.000; Control-Exercise + DM: 0.000; Exercise-DM: 0.000; Exercise-Exercise + DM: 0.022; DM-Exercise + DM: 0.000. The sample size was identical across all experimental groups (*n* = 10 animals per group) for all assays.

**Figure 5:**
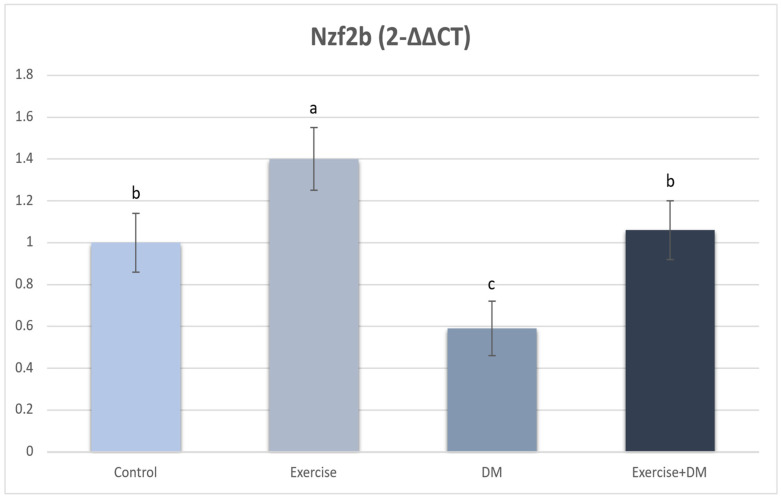
Hippocampal *Nzf2b* gene expressions. Results are presented as mean ± SEM. There are statistically significant differences between groups labeled with different letters (*p* < 0.05; a > b > c). GAPDH was used as an endogenous control. *p*-Values NZB2b: Control-Exercise: 0.000; Control-DM: 0.000; Control-Exercise + DM: 0.767; Exercise-DM: 0.000; Exercise-Exercise + DM: 0.000; DM-Exercise + DM: 0.000. The sample size was identical across all experimental groups (*n* = 10 animals per group) for all assays.

Analysis of biochemical markers revealed that Nogo-A levels were significantly elevated in the G3 group, whereas KLOTHO and NZF-2b levels were significantly reduced (*p* < 0.05). Chronic exercise in diabetic rats (G4) partially reversed these alterations, leading to reduced Nogo-A expression and significantly increased KLOTHO and NZF-2b levels (*p* < 0.05). However, when comparing the values in G4 with the control groups (G1 Control and G2 Exercise), klotho gene expression was found to be higher than in G1 (*p* < 0.05) and lower than in G2 (*p* < 0.05), while nogo-a gene expression was lower than in G1 (*p* < 0.05) and higher than in G2 (*p* < 0.05). NZB2b gene expression was lower than in G2 (*p* < 0.05) and not different from G1 (Figure 3, Figure 4 and Figure 5).

**Figure 6:**
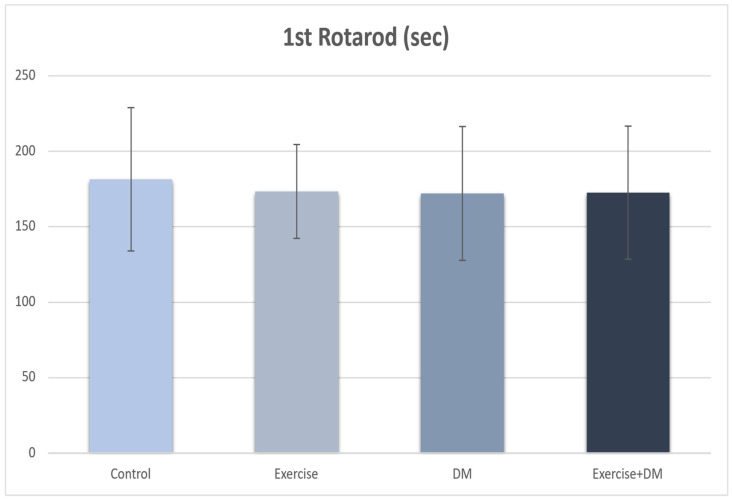
Base rotarod data prior to experimental applications. Results are presented as mean ± SEM. No statistically significant difference was found between the groups in terms of time spent on the bar. The unit is presented as second. *p*-Values Rotarod Baseline: Control-Exercise: 0.970; Control-DM: 0.952; Control-Exercise + DM: 0.960; Exercise-DM: 1000; Exercise-Exercise + DM: 1000; DM-Exercise + DM: 1000. The sample size was identical across all experimental groups (*n* = 10 animals per group) for all assays.

**Figure 7:**
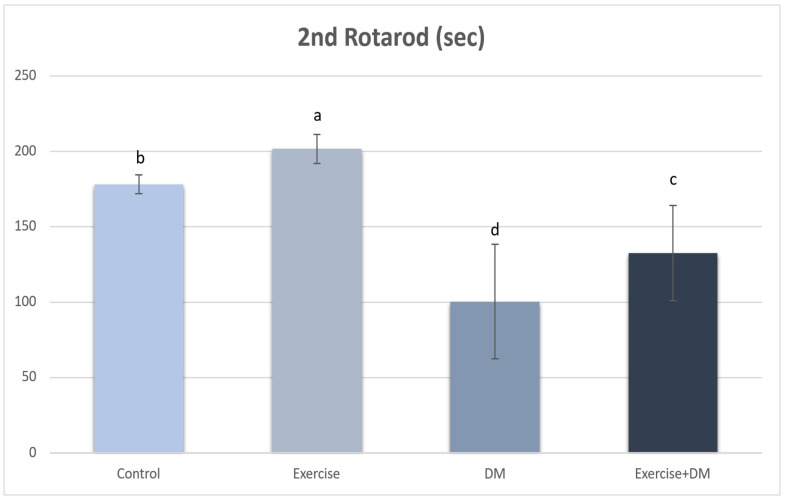
Rotarod data at the end of the experiment. Results are presented as mean ± SEM. There are statistically significant differences between groups labeled with different letters (*p* < 0.05). The unit is presented as second. *p*-Values Rotarod Finish: Control-Exercise: 0.036; Control-DM: 0.000; Control-Exercise + DM: 0.000; Exercise-DM: 0.000; Exercise-Exercise + DM: 0.000; DM-Exercise + DM: 0.005. The sample size was identical across all experimental groups (*n* = 10 animals per group) for all assays.

Evaluation of motor function and behavioral performance showed that the shortest rotarod latency was recorded in the G3 group (*p* < 0.05). In terms of motor performance, exercised diabetic rats (G4) showed a significant increase in motor skills compared to the G3 group (*p* < 0.05). However, the improvement in motor performance observed in the G4 group was below the levels of the control groups (G1 Control and G2 Exercise) (*p* < 0.05; Figure 6 and Figure 7).

## 3. Discussion

In this section of our discussion, we compared diabetes-induced oxidative damage in hippocampal tissue in aged female rats and the protective effects of 4 weeks of chronic treadmill exercise on this damage. Overall, our findings show that diabetes (G3) triggers lipid peroxidation by increasing malondialdehyde (MDA) levels in hippocampal tissue and weakens antioxidant defense by depleting glutathione (GSH) stores. However, the daily 45 min exercise intervention (G4) significantly improved hippocampal tissue damage. The high MDA levels observed in the hippocampus of diabetic rats (G3) are indicative of damage to cellular membranes caused by reactive oxygen species (ROS) induced by chronic hyperglycemia [22]. It is known that diabetes increases mitochondrial dysfunction in brain tissue and the resulting oxidative stress accelerates neurodegenerative processes [23]. The hippocampus, in particular, is a region highly susceptible to metabolic disorders. Our findings are fully consistent with previous studies reporting increased tissue damage in diabetic models [24]. The key finding of our study is that 4 weeks of chronic running exercise (G4) helps restore redox balance in diabetic rats by lowering MDA levels and raising GSH levels. Exercise creates a “hormetic” effect by upregulating the body’s endogenous antioxidant systems through low-dose ROS production [25]. Aerobic exercise, such as treadmill exercise, has been reported in many studies to increase GSH synthesis through various molecular pathways and protect hippocampal neurons from oxidative damage [25,26]. In our study, it can be said that a 45 min daily moderate-intensity exercise protocol for one month provided sufficient stimulus to activate antioxidant defense mechanisms in aged hippocampal tissue. However, the fact that the improvement in the exercise group (G4) did not fully reach the control values (G1 and G2) demonstrates the depth of damage caused by the combined effects of aging and diabetes. It is known that the antioxidant response in elderly individuals is slower and more limited than in younger individuals [27]. Furthermore, the literature discusses that a 4-week period is insufficient to completely eliminate long-term chronic damage from diabetes; protocols of 8 weeks or more are likely necessary for a more significant improvement [28]. In conclusion, chronic treadmill exercise demonstrated a neuroprotective effect by improving hippocampal oxidative stress parameters in aged diabetic rats. These findings support the idea that exercise may be a powerful non-pharmacological tool in preventing diabetes-related cognitive decline.

Our findings, showing increased hippocampal Nogo-A expression in diabetic aged female rats (G3) and that chronic exercise partially reduced it, point to neuroplasticity mechanisms consistent with the literature [29]. The significant increase in Nogo-A in the diabetic G3 group (*p* < 0.05) suggests that hippocampal plasticity and nerve extension regeneration are suppressed in diabetes. Nogo-A, as a myelin-associated growth inhibitor, inhibits axonal elongation and plasticity; similar upregulation is observed in the retina and pancreas in diabetic models [30]. Increased Nogo-A is also associated with impaired glucose metabolism and neurodegenerative processes [31]. Although decreased hippocampal Nogo-A mRNA in old age has been reported in the literature, it appears that diabetes reverses this effect by increasing inhibition [30]. Again, in our study, chronic exercise triggered a neuroprotective response in diabetic rats (G4) by significantly reducing Nogo-A. In the diabetic G4 group, the reduced Nogo-A we obtained with chronic exercise did not reach the control (G1–G2) values. This shows that chronic exercise partially improves the increased Nogo-A levels in the elderly hippocampus. The report that exercise improves plasticity in diabetic rats by preventing hippocampal damage and increasing factors such as BDNF is partially consistent with the findings in our study [32]. The Nogo-A findings we obtained in our study suggest that chronic exercise can partially restore hippocampal plasticity in elderly patients with diabetes; however, combined treatments are needed for complete recovery. It is supported in neurodegenerative models that diabetes accelerates cognitive decline by increasing Nogo-A, and that exercise slows this down [33].

In this section of our discussion, we investigated the effect of chronic exercise on hippocampal Klotho levels in aged diabetic female rats; we found that diabetes suppresses Klotho expression, but regular exercise significantly limits this decrease. The low Klotho levels observed in the G3 group of diabetic aged rats in our study are consistent with diabetic models in the literature. Klotho’s inhibitory effect on the insulin/IGF-1 signaling pathway is considered a mechanism associated with longevity and cellular stress resistance, while Klotho deficiency leads to disruption of metabolic homeostasis at the organismal level, increased oxidative stress, and insulin resistance. Therefore, the data obtained in this study can be interpreted as reflections of Klotho’s multifaceted biological functions at different levels [34]. Chronic high glucose, which is the main marker of diabetes, suppresses Klotho gene synthesis by accumulating advanced glycation end products [35]. The fact that this is more pronounced in aged rats suggests that the naturally decreasing Klotho levels with aging, combined with diabetic inflammation, create a double blow in hippocampal tissue. The findings obtained in the G4 group revealed that chronic exercise significantly increased hippocampal Klotho expression despite diabetic damage. Findings in the literature suggest that physical activity may contribute to the regulation of hippocampal Klotho expression via myokines such as irisin, which are of skeletal muscle origin and can cross the blood–brain barrier.y patients [36]. Increased Klotho enhances synaptic plasticity by optimizing NMDA receptor functions in hippocampal neurons and slows down cognitive decline caused by diabetes [37]. In our study, the failure of the increase in the G4 group, which underwent chronic exercise, to fully reach control levels (G1, G2) reflects the depth of structural hippocampal damage induced by diabetes. Many studies have reported that exercise improves metabolic parameters, especially in elderly subjects, but cannot completely reverse the basal damage caused by chronic pro-inflammatory cytokines (IL-6, TNF-α) [38]. This may indicate that exercise dosage (intensity and duration) is insufficient for “complete reversal” in the elderly and diabetic population, or that epigenetic modifications are persistent. The data from our study confirm that Klotho is a critical molecular target that responds to exercise. This partial improvement provided by exercise is critical for the preservation of cognitive and vital functions at the hippocampus level, even if complete recovery cannot be achieved in diabetic elderly patients.

The present findings indicate that hippocampal NZF-2β gene expression, which is markedly suppressed in diabetic aged female rats, is partially restored by chronic exercise. These results suggest that diabetes downregulates this brain-specific transcription factor, whereas exercise exerts a neuroprotective modulatory effect. In the diabetic aged female group (G3), hippocampal NZF-2β levels were significantly reduced, a change that may be associated with diabetes-induced oxidative stress and neuroinflammation. Previous studies, which parallel the current findings, have reported a decrease in the expression of zinc transporter proteins such as ZnT3 in the hippocampus of diabetic rats [39]. NZF-2β, a brain-specific C2HC-type zinc finger transcription factor also called myelin transcription factor 1 (Myt1) or 7ZFMyt1, plays a critical role in the regulation of neuronal gene expression. Its suppression under diabetic conditions may contribute to impaired transcriptional control associated with metabolic and neurodegenerative processes [4,40]. Notably, chronic exercise (G4 group) significantly increased hippocampal NZF-2β expression in diabetic rats (*p* < 0.05), although expression levels did not fully return to those of the control group. This partial recovery is in line with previous evidence showing that chronic exercise attenuates oxidative damage in the diabetic hippocampus, reduces lipid peroxidation markers such as MDA, and partially restores ZnT3 expression [39]. Together, these findings support the concept that exercise modulates neural transcriptional mechanisms under diabetic conditions. Exercise is also known to enhance cognitive function in diabetes models through upregulation of BDNF and other neurotrophic factors [41]. Therefore, the exercise-induced increase in NZF-2β observed in the present study may represent an additional molecular pathway contributing to neuroprotection. However, the incomplete normalization of NZF-2β expression suggests that factors such as advanced age and female gender may limit the full restorative capacity of exercise in this model. Given the zinc finger structure of NZF-2β, changes in zinc homeostasis may be a significant component of the diabetes-exercise interaction [42]. Supporting this possibility, zinc supplementation has been shown to reduce muscle atrophy in diabetic models [43], suggesting that zinc-dependent regulatory pathways play a broader role in diabetes-related tissue dysfunction. Future studies should therefore explore NZF-2β–related signaling pathways, including potential interactions with BDNF regulation, within the framework of zinc homeostasis, diabetes, and brain function.

No significant differences were found between the groups in the rotarod tests conducted at the beginning of our study, i.e., before the applications to the animals began. This will also allow for a reliable discussion of the rotarod test results measured after the application. In the present study, the findings obtained from the rotarod test demonstrate the detrimental effects of diabetes on hippocampal function and motor learning. The poorest rotarod performance observed in the diabetic aged female rat group (G3) indicates a marked impairment in motor coordination and learning ability. In contrast, diabetic rats subjected to moderate chronic exercise showed a significant improvement in performance; however, their performance did not fully reach control levels. These results confirm the combined negative impact of diabetes and aging on motor performance. The adverse effects of diabetes on the central nervous system have been widely reported in the literature. In streptozotocin (STZ)-induced diabetes models, reductions in hippocampal neuron number and impairments in learning and memory performance have been documented, indicating that diabetes directly affects brain structure and function [44]. Moreover, increased levels of stress markers such as nitrotyrosine and inflammation under chronic diabetic conditions have been associated with molecular alterations that disrupt learning and memory processes [45]. Exercise is a powerful neuroprotective intervention capable of partially reversing the negative effects of diabetes. Studies investigating the relationship between exercise and the nervous system have shown that physical activity enhances cortical and hippocampal neuroplasticity, elevates levels of neurotrophic factors—particularly brain-derived neurotrophic factor (BDNF)—and promotes the strengthening of synaptic connections [46]. In another study conducted in diabetic models, aerobic exercise was reported to increase hippocampal BDNF and synaptic proteins, changes that correlated with improvements in learning and memory performance [47]. These reports are consistent with our findings and further support the positive contribution of exercise to neuroplasticity. The improvement in motor learning performance observed in exercised diabetic animals in our study may therefore be attributed to the neuroplasticity-enhancing effects of exercise. Previous reports have shown that exercise promotes neuroblast differentiation and improves dendritic structure in diabetic models, thereby increasing synaptic formation and synaptic connection potential [48]. However, some studies have shown that the restorative capacity of exercise may be limited, particularly in the chronic stages of diabetes [49]. This may explain why exercise resulted in only partial rather than complete recovery in diabetic models; this is consistent with our finding that the G4 group did not fully reach control levels. In terms of hippocampal tissue integrity, chronic diabetes is known to impair antioxidant defense systems and increase tissue damage. Exercise has been shown to reduce such damage by modulating the oxidative stress response [50]. Consistent with this information, the improvement in hippocampal integrity markers observed in our study can be attributed to the antioxidant and anti-inflammatory effects of exercise. Overall, our rotarod findings demonstrate that exercise supports hippocampal integrity, motor learning, and molecular pathways related to neuroplasticity in an aged diabetic rat model. However, the limited ability of exercise to bring about complete recovery suggests that, due to the chronic nature of the disease, the destructive processes associated with diabetes continue at the synaptic and cellular levels.

## 4. Materials and Methods

### 4.1. Experimental Animals and Ethical Approval

Sixteen-month-old female Wistar rats obtained from the Experimental Medicine Research and Application Center of Selçuk University (SÜDAM) were used in the study. All experimental procedures were approved by the Selçuk University Animal Experiments Ethics Committee (Decision No: 2022-27, 29 July 2022). Analyses were carried out in the Molecular Physiology Laboratory, Department of Physiology, Faculty of Medicine, Selçuk University.

Animals were housed under a 12 h light/12 h dark cycle at an ambient temperature of 21 ± 1 °C, with ad libitum access to standard pellet chow and water.

#### Experimental Groups

Older female rats were used in the experiments. The animals were selected from those that were no longer used for breeding at our institution’s animal testing center due to reduced reproductive capacity. The lower levels of hormonal variability due to decreased reproductive function in older age were a factor in choosing these animals.

A total of 40 rats were randomly divided into four groups (*n* = 10 per group):

Group 1 (G1, Contro): Control—No intervention (*n* = 10);
Group 2 (G2, Exercise): Exercise—Chronic treadmill exercise for 4 weeks (*n* = 10);
Group 3 (G3, DM): Diabetes—Diabetes induced by STZ (*n* = 10);
Group 4 (G4, Exercise + DM): Diabetes + Exercise—Exercise for 4 weeks following STZ induction (*n* = 10).

### 4.2. Induction of Experimental Diabetes

Diabetes was induced in Groups 3 and 4 by a single intraperitoneal injection of 40 mg/kg streptozotocin (STZ; Sigma, S-0130, St. Louis, MO, USA). Seventy-two hours after injection, animals with blood glucose levels ≥ 300 mg/dL measured from the tail vein were considered diabetic (Lifecheck Smart TD-4360 glucometer, Bursa, Türkiye) [51]. Diabetes was successfully induced in all animals treated with STZ (100% success rate), and no animal was observed without developing diabetes.

### 4.3. Chronic Treadmill Exercise Protocol

Running exercises in animals were performed on a multi-channel rat treadmill manufactured by Selçuk University Experimental Medicine Research and Application Center (SUDAM) and located within the center. The device, which allows eight animals to exercise simultaneously, has technical equipment that allows for speed adjustment, time control, and the application of low-intensity electrical impulses to encourage animals to run when necessary. The exercise protocol was started 48 h after confirmation of diabetes onset and was applied for 4 weeks. To ensure the animals’ adaptation to the treadmill, acclimatization exercises were performed for two days prior to the experimental exercise program, for 15 min a day at a speed of 15 m/min. Following the adaptation period, the animals were run for 45 min a day at a speed of 20 m/min, 7 days a week, for 4 weeks. To ensure the animals continued exercising during running, a low-intensity electrical impulse system located at the rear of the treadmill was used when necessary. The exercise duration was planned to minimize the stress effects that long-term running might cause [52]. No animals were observed to have failed to adapt to running exercises during the adaptation process, and no animals were disqualified based on exercise performance.

### 4.4. Rotarod Performance Test

Motor coordination and balance were evaluated using an accelerating rotarod device (May Rotarod, Commat Ltd., Ankara, Turkey). Animals received adaptation training for 3 days before testing. On the test day, the device accelerated from 4 rpm to 20 rpm. The maximum latency to fall was set at 300 s for each animal, and the speed at the time of falling was recorded. During the rotarod tests, all animals completed the test, and no animals were disqualified due to poor performance or non-compliance with the test procedure.

### 4.5. Rat Euthanasia and Collection of Tissue Samples

At the end of the 4-week experimental period, 24 h after the last exercise session, rats were anesthetized with ketamine (75 mg/kg, i.p.) and xylazine (10 mg/kg, i.p.). Sufficient depth of anesthesia was confirmed by toe withdrawal and absence of corneal reflexes. Euthanasia was then performed by cervical dislocation under anesthesia to ensure complete death.

Following euthanasia, brains were rapidly removed, immediately frozen on dry ice, and transferred to Eppendorf tubes. Tissue samples were stored at −80 °C until biochemical analysis. All procedures were performed in accordance with institutional ethical guidelines.

### 4.6. Total RNA Isolation and cDNA Synthesis

Total RNA isolation from hippocampal tissues was performed using a commercial RNA isolation kit (Bio Basic; Markham, ON, Canada) according to the manufacturer’s protocol. The quantity and purity of the obtained RNA were assessed spectrophotometrically (A260/A280 ratio) using a reader (SMA 1000, SonicWall, Shanghai, China). RNA samples with an A260/A280 ratio above 1.9 were considered to be of sufficient quality and were used for cDNA synthesis. cDNA synthesis was performed using a reverse transcription kit (OneScript Plus, ABM, Richmond, BC, Canada).

#### Real-Time Polymerase Chain Reaction (RT-qPCR)

The expression levels of the Nogo-A, KLOTHO, and NZF2b/7ZFMyt1 genes were determined using the real-time polymerase chain reaction (RT-qPCR) method. Amplifications were performed using a SYBR Green-based master mix (BlasTaq 2X qPCR Master Mix; ABM, Canada). Primers were synthesized by Oligomer (Ankara, Turkey) (Table 1). GAPDH was used as the reference gene. All RT-qPCR reactions were performed in triplicate for each biological sample.

### 4.7. Determination of MDA and GSH Levels

For biochemical analyses, hippocampus tissues were homogenized on ice to obtain a 10% tissue homogenate in 150 mM KCl buffer using an ultrasonic homogenizer (BANDELIN SONOPULS mini20, Berlin, Germany). All biochemical measurements were performed in triplicate for each biological sample.

#### 4.7.1. Determination of Malondialdehyde (MDA) Levels

Malondialdehyde (MDA) levels were determined using the Mihara and Uchiyama method as an indicator of lipid peroxidation [54]. Measurements were performed using a SPEC-TROstar microplate reader (BMG Labtech, Ortenberg, Germany). The results obtained are expressed in nmol/g tissue.

#### 4.7.2. Determination of Glutathione (GSH) Levels

Glutathione (GSH) levels in hippocampus tissue were measured based on the Ellman method (Sedlak and Lindsay [55]). Analyses were performed on a SPECTROstar (BMG Labtech, Germany) instrument, and results were presented in mmol/g tissue.

### 4.8. Statistical Analysis

qPCR data were analyzed using the 2^−ΔΔCt^ method [24]. Statistical analyses were performed using SPSS software (version 26.0; IBM Corp., Armonk, NY, USA). Results are presented as mean ± standard error (SEM).

Normality of data distribution was assessed using the Shapiro–Wilk test. Comparisons among groups were performed using one-way analysis of variance (ANOVA), followed by Duncan’s post hoc test for multiple comparisons. A value of *p* < 0.05 was considered statistically significant.

## 5. Conclusions

The findings of the current study demonstrate that diabetes in aged female rats impairs neuroplasticity and motor performance by increasing oxidative stress. Increased Nogo-A and decreased Klotho and NZF-2b levels, along with impaired antioxidant activity in the hippocampus, provide evidence that senile diabetes creates a degenerative molecular environment. Moderate-intensity chronic exercise partially improved motor performance by reducing oxidative damage and promoting neuroplasticity. However, the failure to achieve complete normalization points to persistent neurobiological effects of aging and diabetes, suggesting that aerobic exercise offers a protective approach.

### Limitations

Despite the significant findings of this study, several limitations need to be considered.

First, the study was conducted only on aged female Wistar rats. While this design allowed for the investigation of sex-specific and age-dependent effects, the generalizability of the findings to male subjects and other age groups has not yet been determined.

Second, the exercise intervention was limited to four weeks of moderate-intensity treadmill training. While this duration may be sufficient to induce measurable molecular and behavioral changes, it may not be sufficient to completely eliminate the chronic and cumulative neurobiological damage caused by diabetes and aging.

Third, although Nogo-A, Klotho, and NZF-2b gene expression levels were measured at the mRNA level using RT-qPCR, protein-level validation via immunohistochemistry or Western blot analysis was not performed.

Fourthly, one of the significant limitations of this study is that gene expression analyses were limited to specific genes. While the mechanisms investigated in the current study could include a variety of molecular processes, including neurotrophic signaling pathways, some important markers such as BDNF were not evaluated within the scope of this study. Therefore, the mechanistic explanations proposed in this study should be interpreted in light of the existing literature rather than directly from experimental evidence. Future studies including broader gene panels will contribute to a more detailed elucidation of the biological mechanisms underlying the observed effects.

## Figures and Tables

**Table 1:** Primers used for target genes.

Gene	Primer Sequence
*NOGO-A*	Forward: 5′-TCAAAGGTGACTGAGGCAGC-3′
	Reverse: 5′-ACTGGGCTGCACTACAGA-3′
*KLOTHO*	Forward: 5′-GGCGACTACCCAAAGAGTATG-3′
	Reverse: 5′-CCGAAGGAGAGAGCAAAGAAG-3′
*NZF2b/7ZFMyt1*	Forward: 5′-GCA GAC CTC AGT TGT CCT ACC-3′
	Reverse: 5′-CTT GGA TAC CAG GTG CTC AG-3′
*GAPDH*	Forward: 5′-GGGCCAAAAGGGTCATCATC-3′
	Reverse: 5′-AACCTGGTCCTCAGTGTAGC-3′

Expression levels of target genes were normalized using a reference gene (e.g., GAPDH/β-actin). Relative gene expression was calculated using the 2^−ΔΔCt^ method [53].

## Data Availability

The raw data supporting the conclusions of this article will be made available by the authors on request.
